# The Sodium-Glucose Cotransporter-2 Inhibitor Canagliflozin Alleviates Endothelial Dysfunction Following *In Vitro* Vascular Ischemia/Reperfusion Injury in Rats

**DOI:** 10.3390/ijms22157774

**Published:** 2021-07-21

**Authors:** Sevil Korkmaz-Icöz, Cenk Kocer, Alex A. Sayour, Patricia Kraft, Mona I. Benker, Sophia Abulizi, Adrian-Iustin Georgevici, Paige Brlecic, Tamás Radovits, Sivakkanan Loganathan, Matthias Karck, Gábor Szabó

**Affiliations:** 1 Department of Cardiac Surgery, University Hospital Heidelberg, 69120 Heidelberg, Germany; cenk.kocer@web.de (C.K.); alexali.sayour@gmail.com (A.A.S.); kraft@uni-heidelberg.de (P.K.); m.i.benker@t-online.de (M.I.B.); sophiaabulizi@yahoo.de (S.A.); paigebrlecic@yahoo.com (P.B.); sivakkanan@gmail.com (S.L.); matthias.karck@med.uni-heidelberg.de (M.K.); Gabor.Szabo@uk-halle.de (G.S.); 2 Heart and Vascular Center, Semmelweis University, 1122 Budapest, Hungary; radovitstamas@yahoo.com; 3 Department of Anesthesiology, St. Josef Hospital, Ruhr-University Bochum, 44791 Bochum, Germany; igeorgevici@outlook.com; 4 Department of Cardiac Surgery, University Hospital Halle (Saale), 06120 Halle, Germany

**Keywords:** ischemia/reperfusion, endothelial function, canagliflozin, sodium-glucose cotransporter-2, diabetes mellitus

## Abstract

Vascular ischemia/reperfusion injury (IRI) contributes to graft failure and adverse clinical outcomes following coronary artery bypass grafting. Sodium-glucose-cotransporter (SGLT)-2-inhibitors have been shown to protect against myocardial IRI, irrespective of diabetes. We hypothesized that adding canagliflozin (CANA) (an SGLT-2-inhibitor) to saline protects vascular grafts from IRI. Aortic rings from non-diabetic rats were isolated and immediately mounted in organ bath chambers (control, n = 9–10 rats) or underwent cold ischemic preservation in saline, supplemented either with a DMSO vehicle (IR, n = 8–10 rats) or 50µM CANA (IR + CANA, n = 9–11 rats). Vascular function was measured, the expression of 88 genes using PCR-array was analyzed, and feature selection using machine learning was applied. Impaired maximal vasorelaxation to acetylcholine in the IR-group compared to controls was significantly ameliorated by CANA (IR 31.7 ± 3.2% vs. IR + CANA 51.9 ± 2.5%, *p* < 0.05). IR altered the expression of 17 genes. *Ccl2*, *Ccl3*, *Ccl4*, *CxCr4*, *Fos*, *Icam1*, *Il10*, *Il1a* and *Il1b* have been found to have the highest interaction. Compared to controls, IR significantly upregulated the mRNA expressions of *Il1a* and *Il6*, which were reduced by 1.5- and 1.75-fold with CANA, respectively. CANA significantly prevented the upregulation of Cd40, downregulated NoxO1 gene expression, decreased ICAM-1 and nitrotyrosine, and increased PECAM-1 immunoreactivity. CANA alleviates endothelial dysfunction following IRI.

## 1. Introduction

Coronary artery bypass grafting (CABG) with autologous conduits remains one of the most frequently performed procedures to restore blood flow to ischemic myocardium. The harvest and perioperative storage of these vascular grafts results in endothelial dysfunction, which is further exacerbated by ischemia/reperfusion (IR) injury (IRI) upon implantation and restoration of blood flow. The reperfusion of previously ischemic tissue is accompanied by the rapid restoration of a physiologic pH, calcium overload, ATP depletion, neutrophil influx, the generation of reactive oxygen species (ROS), the release of pro-inflammatory cytokines, and the enhanced expression of adhesion molecules, ultimately leading to vascular damage [1]. These events contribute to bypass graft failure, jeopardizing long-term clinical outcomes [2]. Therefore, there is an urgent need for the development of novel storage solutions to prolong vascular graft integrity and enhance long-term graft patency [3,4].

Canagliflozin (CANA) is a member of a novel class of antidiabetic agents, selective sodium glucose-cotransporter (SGLT)-2 inhibitors, which have been approved for the treatment of patients with type-2 diabetes mellitus (T2DM) [5]. These medications reduce the kidneys’ glucose reuptake, increasing urinary glucose excretion and thus lowering blood sugar levels [6]. The Canagliflozin Cardiovascular Assessment Study (CANVAS) Program [7] and CREDENCE trials [8] were designed to examine the potential cardiovascular effects of CANA. Interestingly, these trials have demonstrated reductions in the primary composite endpoint of cardiovascular death, nonfatal myocardial infarction, and nonfatal stroke compared to placebo and standard care in patients with T2DM [9]. Furthermore, two recent clinical trials (EMPEROR-Reduced [10] and DAPA-HF [11]) support the use of SGLT2 inhibitors for the treatment of patients with heart failure and a reduced ejection fraction, regardless of the presence or absence of diabetes. Additionally, it has been shown that short- or long-term administration of CANA attenuates myocardial IRI in diabetic and nondiabetic rats [12,13,14]. These findings support the hypothesis that CANA might also protect against vascular IRI, irrespective of diabetic status and raise the possibility of repurposing the SGLT-2 inhibitor as a novel cardioprotective intervention in high-risk nondiabetic patients with significant pre-existing cardiovascular disease. Indeed, we have recently shown that CANA has direct vasoactive properties in aortic rings from nondiabetic, healthy rats [14]. A recent *in vitro* study has shown that CANA conveys direct anti-inflammatory actions in lipopolysaccharide-stimulated human coronary artery endothelial cells [15]. Furthermore, Mancini et al. have demonstrated that in cultured endothelial cells CANA inhibited key proinflammatory cytokine secretion, such as IL-6 and the chemokine monocyte chemoattractant protein (MCP)-1 (also known as CCL2) [16]. The increased expression of IL-6 recruits circulating leukocytes to the vascular wall by up-regulating chemokine production and adhesion molecule expression, contributing to endothelial dysfunction in part by increasing vascular superoxide and limiting nitric oxide (NO) bioavailability. Additionally, CANA has been shown to prevent diabetes-induced endothelial dysfunction in ApoE-deficient mice through its anti-inflammatory and antioxidative potential [17].

As the direct impact of CANA on IR-induced vascular damage has not been previously investigated, we hypothesized that physiological saline supplemented with CANA protects grafts from *in vitro* vascular IRI.

## 2. Results

### 2.1. Aortic Vasoreactivity Following Vascular IRI

#### 2.1.1. Effect of CANA on Endothelial Function after Vascular IRI

**Figure 1:**
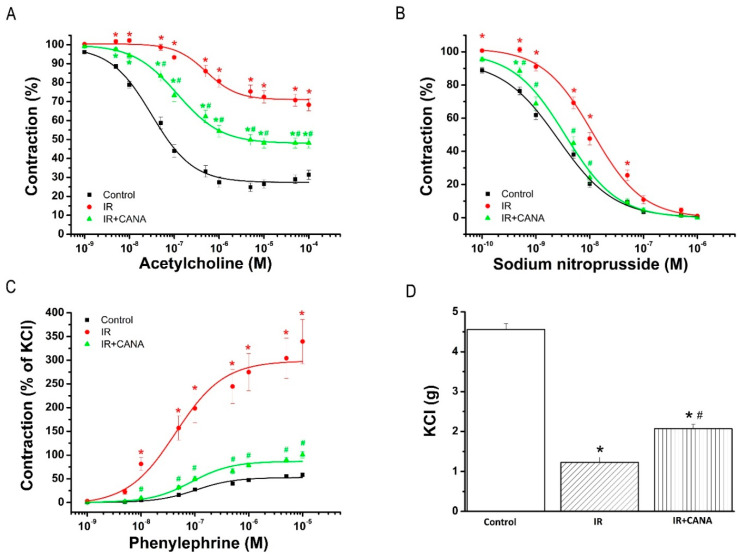
Effect of canagliflozin (CANA) on contractile and relaxation responses after vascular ischemia/reperfusion (IR) injury. (**A**) Acetylcholine-induced endothelium-dependent and (**B**) sodium nitroprusside-induced endothelium-independent vasorelaxation, and contractile responses (**C**) to phenylephrine (percentage of the maximum contraction induced by potassium chloride (KCl)) and (**D**) to high potassium-induced depolarization of isolated aortic rings. Results are represented as mean ± SEM. * *p* < 0.05 versus Control; ^#^ *p* < 0.05 versus IR. n = 18–43 aortic rings from 8–11 rats.

Acetylcholine (ACh, 10^−9^–10^−4^ M) induced concentration-dependent relaxation in aortic rings precontracted with phenylephrine (PE) in all experimental groups (Figure 1A). Reduced endothelium-dependent vasorelaxation in response to ACh in the IR group compared to controls was significantly improved by the preservation of aortic rings with CANA (Table 1, Figure 1A). Furthermore, decreased aortic sensitivity (pD_2_-value) to ACh seen after IRI was significantly ameliorated by CANA (Table 1).

#### 2.1.2. Effects of CANA on Smooth Muscle Relaxation after Vascular IRI

Sodium nitroprusside (SNP, 10^−10^–10^−5^ M), an endothelium-independent vasodilator, evoked a concentration-dependent relaxation of PE precontracted aortic segments (Figure 1B). Although there was no difference in maximal relaxation to SNP, the concentration-response curve from the IR + CANA group was significantly left-shifted compared to that of the IR group (Table 1, Figure 1B).

#### 2.1.3. Effects of CANA on Contractility after Vascular IRI

Increased maximal PE-induced contractile response in the IR group compared to controls was significantly decreased via the preservation of aortic rings with CANAs (Table 1, Figure 1C). Increased sensitivity (pD_2_-value) to PE in the IR rings compared to controls was significantly reduced by CANA (Table 1). Decreased maximal 80 mM K^+^-induced contraction in the IR group compared to controls was significantly increased by CANA (Figure 1D).

### 2.2. Effects of CANA on Aortic Gene Expression Following Vascular IRI

**Figure 2:**
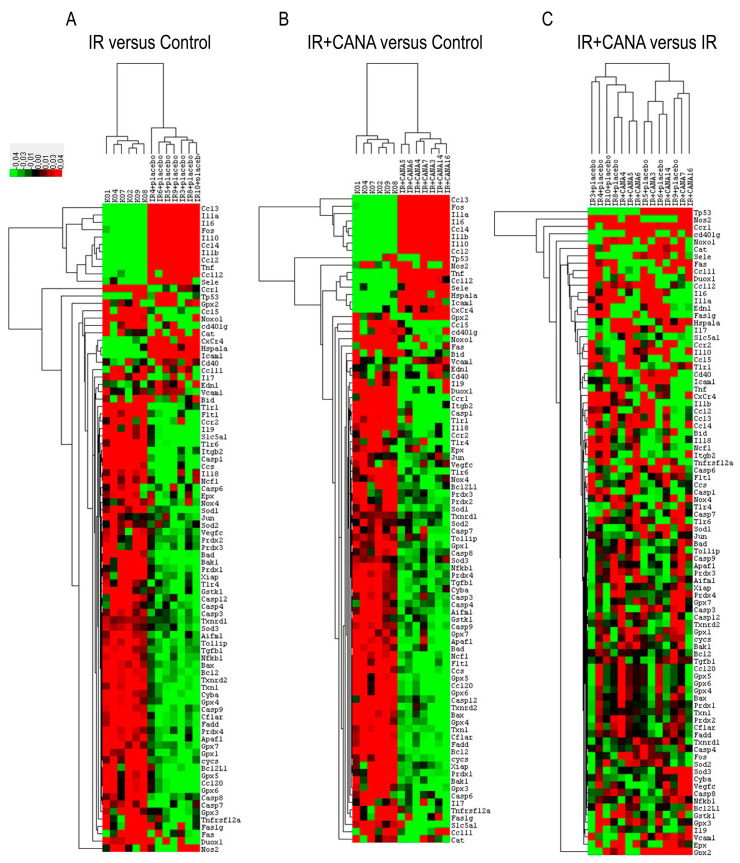
Changes in gene expression caused by vascular ischemia/reperfusion (IR) injury and the effect of canagliflozin (CANA). The expression of 88 genes involved in inflammation, apoptosis, and oxidative stress has been profiled. Clustergrams create a heat map with dendograms to indicate which genes are coregulated. Degrees of red and green indicate relatively high and low expression of the corresponding gene, respectively, and black squares denote genes equally expressed. (**A**) IR vs. Control, (**B**) IR + CANA vs. Control, and (**C**) IR + CANA vs. IR groups. The *x*-axis indicates number of rats (“KO” corresponds to the Control group, including six rats (KO1, KO2, KO4, KO7, KO8, KO9), “IR + placebo” corresponds to the IR group, including seven rats (IR3 + placebo, IR4 + placebo, IR5 + placebo, IR6 + placebo, IR8 + placebo, IR9 + placebo, IR10 + placebo), and “IR + CANA” corresponds to the IR + canagliflozin group, including seven rats (IR + CANA3, IR + CANA4, IR + CANA5, IR + CANA6, IR + CANA7, IR + CANA14, IR + CANA16)) and the *y*-axis indicates the genes. n = 6–7 rats/group. See Table 1 for genes’ abbreviations.

To determine the effects of CANA on IRI-induced vascular changes, the expression levels of 88 genes involved in oxidative stress, apoptosis, and inflammation were simultaneously surveyed using RT^2^ polymerase chain reaction (PCR) Profiler. Among them, the expression of 17 genes was significantly altered in the IR group compared to controls (16 genes were upregulated: *Ccl12*, *Ccl2*, *Ccl3*, *Ccl4*, *Cd40*, *Cxcr4*, *Edn1*, *Fos1*, *Hspa1a*, *Icam1*, *Il10*, *Il1a*, *Il1b*, *Il6*, *Sele*, *Tnf* and 1 gene was downregulated: *Ccl5*, Table 2, first column). Compared to the controls, the upregulated mRNA expression of *Il1a* and *Il6* in the IR rings was reduced 1.5- and 1.75-fold with CANA, respectively. Furthermore, CANA prevented the upregulation of *Cd40* and significantly downregulated *NoxO1* gene expression compared to controls (Table 2, second column). Figure 2 shows clustergram analysis and heat map graphs of gene expression data.

### 2.3. Aortic Gene Expression Explored by Machine Learning Algorithms

**Figure 3:**
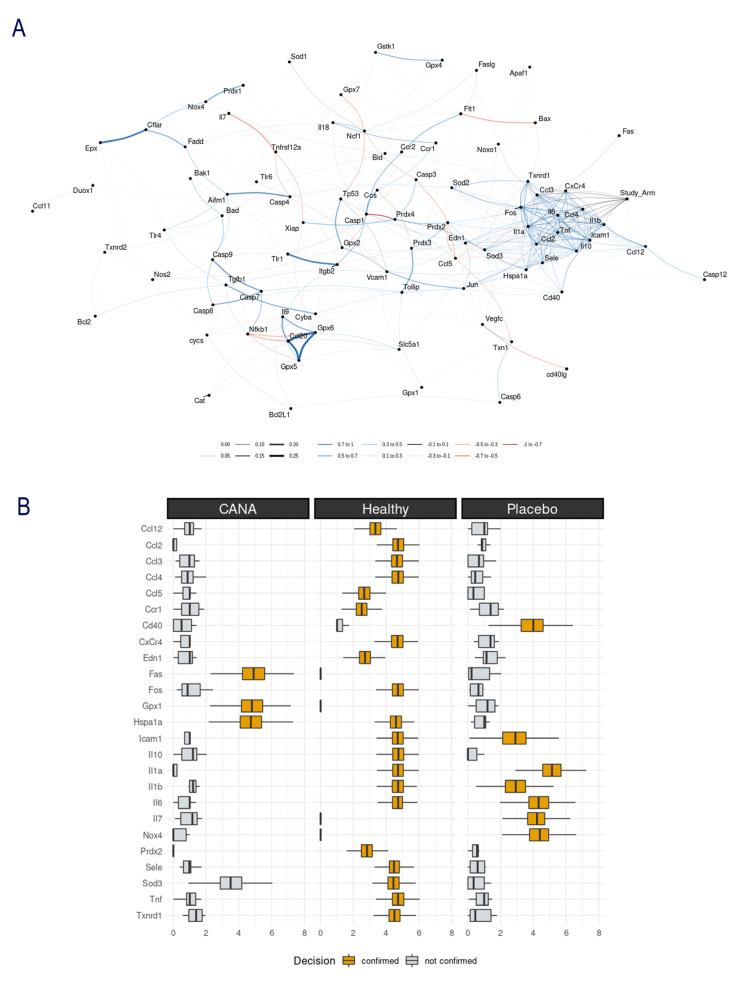
Machine-learning analysis: (**A**) Gene correlation network: a node represents each investigated gene. The closeness between nodes and the connection width are proportional to the random-forest variable importance. The colors indicate the Spearman’s coefficients: blue or red lines are associated with a positive or negative correlation, respectively. The grey connections are random-forest patterns without a significant Spearman coefficient. “Study arm” is the group variable: Control/IR/IR + CANA. This node is connected (right side of the network), with a cluster of genes, including *Txnrd1*, *Ccl3*, *CxCr4*, *Il6*, *Ccl4*, *Il1b*, *Tnf*, *Ccl2*, *Il10*, *IL1a*, *Fos*, *Sod3*, *Hspa1a*, *Cd40*, *Ccl12* and *Icam1*. These genes are in a significant positive Spearman correlation with each other. In contrast, the other tested genes do not show a cluster pattern. (**B**) Genes predicting the experimental group (Control, IR, or IR + CANA). The genes identified with the yellow horizontal box plots respond to the question: “is this IR + CANA or not?”, “is this Control or not?”, “is this IR or not?”. The *Y*-axis depicts genes (abbreviations are defined in Table 1) and the horizontal axis indicates the predictive power of a gene for the group identification, as calculated by the random forest model. IR indicates ischemia/reperfusion, CANA canagliflozin.

A computational analysis using Crossboruta was performed on the gene expression profiles. Figure 3A displays the network of genes predictive for inter-group classification. The right portion of the network graph, observes that the node “study arm” (Control/IR/IR + CANA) is connected to a cluster of positively intercorrelated genes. The periphery shows *Ccl3*, *CxCr4*, *Ccl4*, *Il1b*, *Icam1*, *Il10*, *Ccl2*, *Il1a* and *Fos*. The cluster’s centroid contains the Il6 and Tnf, suggesting that these two might play a central role in IRI’s pathophysiology. Figure 3B depicts the random-forest based gene selection; the variables confirmed as predictive in the group classification are *Fos*, *Gpx1* and *Hspa1a* in the IR + CANA rings and *Cd40*, *Icam1*, *Il10*, *Il1b*, *Il6*, *Il7* and *Nox4* in the IR group. *Il1a*, *Il6*, *Cd40* and *Icam1* were not predictive for the IR + CANA group but were predictive for the IR aortic rings (Figure 3B).

### 2.4. Effects of CANA on Aortic Intercellular Adhesion Molecule (ICAM)-1, Platelet Endothelial Cell Adhesion Molecule (PECAM)-1, Nitrotyrosine, and Caspase-3 Immunoreactivity after Vascular IRI

**Figure 4:**
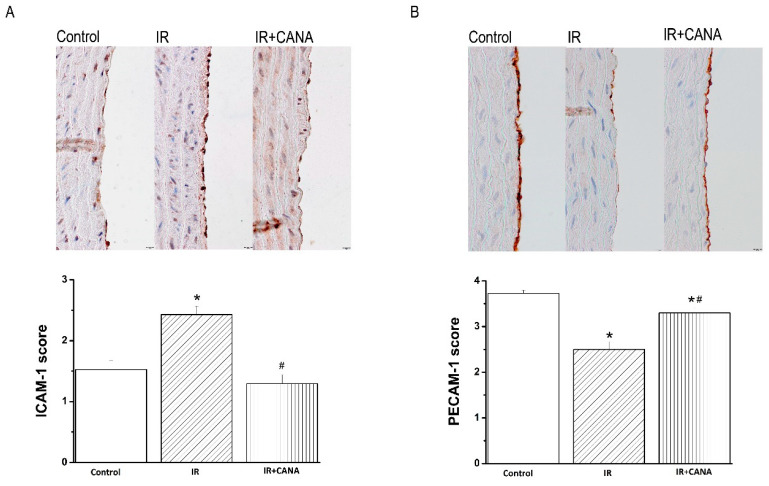
Effect of canagliflozin (CANA) on aortic intercellular adhesion molecule (ICAM)-1 and platelet endothelial cell adhesion molecule (PECAM)-1/CD31 expression after vascular ischemia/reperfusion (IR) injury. Semiquantitative scoring of (**A**) ICAM-1 and (**B**) PECAM-1 immunohistochemical staining with representative micrographs (×400, scale: 10 µm). Results are represented as mean ± SEM. * *p* < 0.05 versus Control; ^#^ *p* < 0.05 versus IR. n = 28–40 aortic rings from 7–10 rats.

Immunohistochemical analysis showed that increased ICAM-1 immunoreactivity in the IR group compared to controls was significantly decreased by preservation of aortic rings with CANA (Figure 4A). Furthermore, decreased PECAM-1 positivity confined to the endothelial layer in the IR rings compared to controls was significantly increased after CANA treatment (Figure 4B).

**Figure 5:**
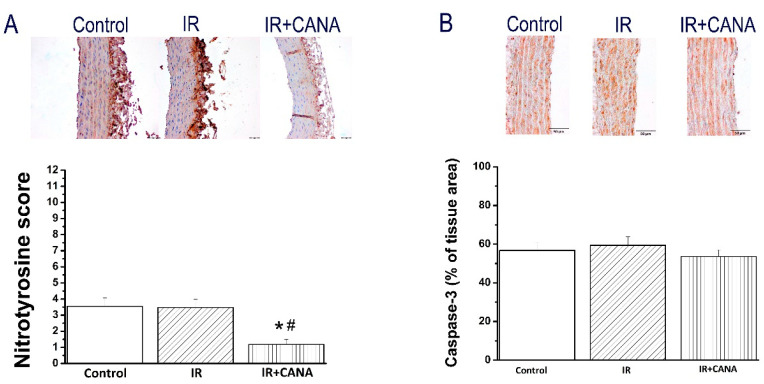
Effect of canagliflozin (CANA) on aortic nitrotyrosine and caspase-3 expression after vascular ischemia/reperfusion (IR) injury. (**A**) Semiquantitative scoring of nitrotyrosine and (**B**) quantitative analysis of caspase-3 immunohistochemical staining with representative micrographs (scale: 50 µm). Results are represented as mean ± SEM. * *p* < 0.05 versus Control; ^#^ *p* < 0.05 versus IR. n = 32–44 aortic rings from 8–11 rats.

Additionally, immunohistochemical data for nitrotyrosine showed lower brown staining in the aortic rings from IR + CANA compared to both control and IR groups (Figure 5A), whereas caspase-3 immunoreactivity was not different among the groups (Figure 5B).

## 3. Discussion

**Figure 6:**
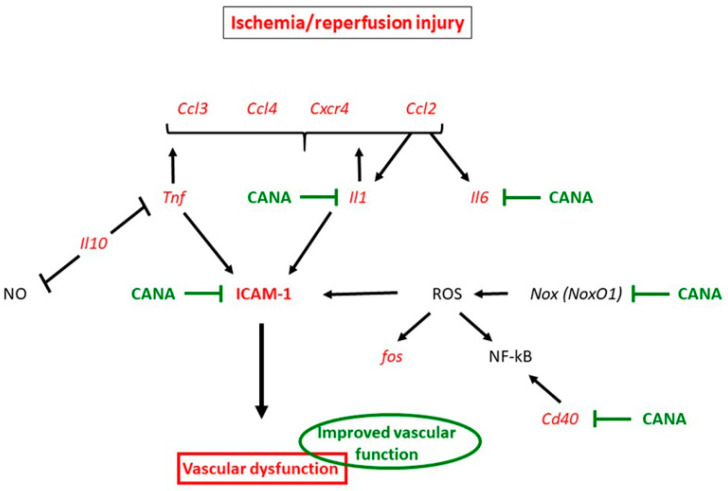
Simplified diagram illustrating the protective effect of canagliflozin (CANA) after vascular ischemia/reperfusion injury (IRI). IRI significantly increased the mRNA expression of *Tnf* and *Il1*, which may increase the expression of *Ccl3*, *Ccl4*, *Cxcr4*, *Ccl2* and ICAM-1. Additionally, IRI increased *fos* and *Ccl2* expression and may upregulate *Il1* and *Il6*, all leading to vascular dysfunction. CANA reduced ICAM-1 expression possibly via preventing the upregulation of the key proinflammatory mediators *Il1*, *Il6* and *Cd40* and by down-regulating *NoxO1*, thereby alleviating endothelial dysfunction. Effects of IRI are indicated by red colors and CANA is indicated by green colors. See Table 1 for gene abbreviations.

In the present work, we hypothesized that adding CANA, an SGLT2 inhibitor developed for the treatment of hyperglycemia in T2DM, to storage solution (physiological saline), protects the vascular grafts of nondiabetic rats against IRI. To our knowledge, this is the first study to suggest that the preservation of aortic rings with CANA alleviates vascular dysfunction following *in vitro* IRI. CANA could exert protective effects by lowering inflammatory response through ICAM-1, PECAM-1 and genes for *Il1a*, *Il6a*, *NoxO1* and *Cd40*, without leukocyte engagement. A graphic summary of our main findings is provided in Figure 6.

CABG is a surgical procedure performed to restore blood flow to areas of the ischemic myocardium supplied by a coronary artery with stenosis. An autologous vessel is used as a graft in most cases. During harvesting these vessels, vascular damage occurs that has a negative impact on future graft patency, and hence on clinical outcomes following CABG. In the present study, an isolated tissue bath system was used to assess the effects of IRI on vascular functional changes. We have confirmed that IRI decreased both endothelium-dependent vasorelaxation and sensitivity to exogenous ACh, impaired smooth muscle relaxation to SNP (an endothelium-independent vasodilator), impaired contraction produced by high potassium chloride (KCl) concentrations and by an α-adrenergic receptor agonist PE in aortic rings originating from healthy, nondiabetic rats. Evidence implicates that inflammation, oxidative stress, and apoptosis play a critical role in the vascular response to IRI [18]. We profiled the aortic expression 88 genes involved in the above-mentioned pathways and described that IRI altered the expression of 17 genes (*Ccl12*, *Ccl2*, *Ccl3*, *Ccl4*, *Cd40*, *Cxcr4*, *Edn1*, *Fos1*, *Hspa1a*, *Icam1*, *Il10*, *Il1a*, *Il1b*, *Il6*, *Sele* and *Tnf* were upregulated and *Ccl5* was downregulated) compared to controls. The PCR array data was further analyzed with random-forest-based algorithms to gain novel insights. Among the tested 88 genes, *Ccl2*, *Ccl3*, *Ccl4*, *CxCr4*, *Fos*, *Icam1*, *Il10*, *Il1a* and *Il1b* have been found to have higher gene–gene interaction than the others, indicating the biological significance of these genes. Two genes, *Il6* and *Tnf*, were identified to have a central role in IRI’s pathophysiology in our setup. These findings may indicate a relationship between these genes and the cause of vascular dysfunction in aortic rings submitted to IRI from nondiabetic rats. Finally, we performed immunohistochemistry to determine if IRI was associated with cellular changes in the aorta. Our results showed that IRI significantly altered the protein expression levels of major intercellular adhesion molecules such as ICAM-1 and PECAM-1, which play important roles in inflammatory processes of the vasculature.

### 3.1. Mechanisms Underlying Protective Effects of CANA against IRI in Vasculature

In the clinical setting, an important issue is the development of novel preservation solutions/methods to protect endothelial cell function against IRI. The SGLT2 inhibitor CANA, a novel oral antidiabetic agent, has been shown to attenuate IRI in the heart [12,14]. We investigated graft preservation in physiological saline enriched with CANA in an experimental model of vascular dysfunction induced by IRI. The principal novelty is that incubation of aortic rings originating from nondiabetic rats protected against endothelial dysfunction following IRI *ex vivo* (evidenced by improved endothelium-dependent vasorelaxation and increased aortic ring sensitivity to ACh). It has been suggested that the direct inhibition of SGLT-2 in the vascular wall contributes to improvement in endothelial function [19]. In the endothelium, muscarinic ACh receptors trigger NO production via endothelial NO synthase (eNOS), which plays a crucial role in regulating vascular function, local blood flow, leukocyte–endothelial cell interactions, platelet aggregation, and adhesion [20]. Reperfusion subsequent to ischemia leads to an extensive inflammatory response characterized by the enhanced expression of adhesion molecules. PECAM-1/CD31, a multifunctional cell adhesion molecule, has been shown to maintain eNOS activity and NO bioavailability [21] and to regulate the extravasation of leukocytes during inflammatory processes [22]. In line with these observations, we have shown that CANA significantly increased the expression of CD31 in IR aortic rings. Additionally, inflammatory molecule ICAM-1 expression can be upregulated by many cytokines and agonists (such as IL-1, TNF and interferon (IFN)-γ) [23], as well as by cytokine-independent stimuli (such as free radicals and hypoxia) [24]. In the present study, we have found that mRNA expression of *Il1*, *Tnf* and *Icam1* was significantly upregulated by IRI, and CANA lowered ICAM-1 immunoreactivity and reduced the gene expression of pro-inflammatory cytokines *Il1a* and *Il6* by 1.5- and 1.75-fold, respectively. A recent study demonstrated that 8-week CANA treatment (30 mg/kg/day) ameliorated endothelium-dependent vasodilation in diabetic atherosclerotic mouse model [17]. The expression of adhesion molecule ICAM-1 was significantly reduced by CANA in ApoE^−/−^ mice, indicating that CANA attenuated vascular inflammation [17]. In our study, we demonstrated that down-expression of ICAM-1 by CANA in aortic rings submitted to *in vitro* IRI (i.e., in the absence of leucocyte binding) was associated with less endothelial dysfunction. Consistent with our results, Clark et al. have shown that elevated ICAM-1 expression in cultured endothelial cells can cause vascular leakiness, cytoskeletal reorganization, and junctional protein alterations without the contribution of leukocytes [25]. Furthermore, the machine-learning algorithms showed that *Il1a*, *Il6*, *Cd40* and *Icam1* were not predictive for the IR + CANA group, but were predictive for the IR aortic rings, suggesting that CANA modifies the expression of the above-mentioned genes. In line with our observation, Mancini et al. have demonstrated that CANA directly inhibits the secretion of proinflammatory cytokine IL-6 and MCP-1 in cultured human endothelial cells [16]. Moreover, the anti-inflammatory effects of CANA have also been suggested in lipopolysaccharide-stimulated coronary artery endothelial cells [15]. Furthermore, in the present study, the preservation of IR rings with CANA decreased nitrotyrosine immunoreactivity, suggesting reduced nitro-oxidative stress.

In the study of El-Daly et al., myocardial IRI was not associated with alteration in SGLT2 mRNA expression [26]. However, in response to high glucose, the expression of SGLT2 has been shown to decrease in the vasculature [26] and to increase in human renal tubular epithelial cells [27]. Indeed, T2DM is associated with upregulation of renal SGLT2 expression in murine and human kidney [28]. There is also evidence that SGLT2 is not involved in renal IRI, as shown by genetic deletion of SGLT2 in a murine model [29], whereas knock-out of SGLT1 protects against renal IRI [30]. Interestingly, the mRNA expression of SGLT1 was not changed in wild-type mice in IRI, compared with healthy controls. Although in the heart, SGLT2 is not expressed, knock-out of SGLT1 has been shown to reduce myocardial infarct size [31]. This class of drug may exert “off-target” cardiovascular benefits by modulating vascular endothelial cell activation and alleviating endothelial cell dysfunction. This is also supported by the work of Ange et al., who recently showed that CANA activates the alpha-1 AMPK-dependent pathway in human endothelial cells exposed to human septic plasma [32]. Furthermore, Behnammanesh et al. have demonstrated that CANA stimulates the expression of heme-oxygenase (HO)-1, a highly inducible antioxidant, in vascular smooth muscle cells. The induction of HO-1 by CANA requires ROS and is mediated by NF-E2-related factor (Nrf2), which contributes to its cellular actions [8]. Taken together, the ability of CANA to exert pleiotropic vascular protective effects suggests extra potential benefits.

It has been shown that blockade of CD40 ligand and its membrane receptor CD40′s interactions prevent the development of vascular inflammation [33]. In line with these observations, we have shown that CANA prevented the upregulation of *Cd40*. Furthermore, we have demonstrated that in the rat aorta, mRNA expression levels for *NoxO1*, one of components forming NADPH oxidase (NOX)-1, was lowered in the IR + CANA group compared to the IR aortic rings. It is known that NOX and eNOS uncoupling are important vascular sources generating ROS, contributing to reduced NO production and endothelial dysfunction [34]. We recently showed that *in vitro* preincubation of aortic rings with CANA enhanced endothelium-dependent NO mediated vasorelaxation and increased sensitivity to ACh in nondiabetic, healthy rats [14]. A final point of discussion is that decreased contraction produced by high KCl in aortic rings was also augmented after CANA treatment. This result suggests that CANA restores cell membrane depolarization-induced contraction in smooth muscle cells. Additionally, an increased PE-induced contractile response was reduced by CANA, which could be related to improved endothelial function. SNP, an endothelium-independent vasodilator, fully relaxed aortic rings in all groups by direct effect on the smooth muscle cells. In the IR group treated with CANA, the concentration–relaxation curves were shifted leftwards compared with the IR aortic rings. Altogether, these results suggest that CANA protects vascular smooth muscle cells against damage from IRI.

### 3.2. Study Limitations

The current study has some limitations that should be acknowledged. First, the role of surrounding tissues of the aorta, blood flow, and the activation of leukocytes need to be investigated in a relevant *in vivo* situation. Second, additional investigations are required to confirm the effects of IRI and CANA on human internal mammary artery and saphenous vein grafts in patients with and without T2DM undergoing CABG. Third, other relevant genes (such as *Slc5a2*), genes encoding major ion channels, and mitochondrial proteins were not investigated. Additionally, even though it is not common in clinical practice, storage at 4 °C for 24 h is a well-established *in vitro* vascular model for IRI. Finally, machine-learning analysis of gene expression dataset is a powerful new tool; however, a larger samples size is needed for a deep learning approach to predict diseases.

## 4. Materials and Methods

### 4.1. Animals

Three month old, nondiabetic male Wistar rats (Janvier Labs, Saint Berthevin, France) were housed under controlled temperature (22 ± 2 °C) and 12–12 h light-dark cycles rooms with ad libitum access to food and water, and acclimatized for at least 7 days prior to experiments. All animals received humane care in compliance with the ‘Principles of Laboratory Animal Care’, formulated by the National Society for Medical Research, and with the ‘Guide for the Care and Use of Laboratory Animals’, prepared by the Institute of Laboratory Animal Resources and published by the National Institutes of Health (NIH Publication, 8th Edition, 2011) with prior approval (on 5 April 2019) by the appropriate institutional review committees (T20/19).

### 4.2. Rat Model of Vascular IRI

#### 4.2.1. Preparation of Aortic Rings

The rats were euthanized with an overdose of sodium pentobarbital (120 mg/kg, intraperitoneally). The descending thoracic aorta was immediately explanted, carefully freed from periadventitial fat and connective tissue under an operation microscope, placed in a Petri dish containing either cold (+4 °C) oxygenated Krebs–Henseleit solution (KHL) with the following composition (mM): 118 NaCl, 4.7 KCl, 1.2 KH_2_PO_4_, 1.2 MgSO_4_, 1.77 CaCl_2_, 25 NaHCO_3_ and 11.4 glucose, or cold physiological saline solution, and was cut into 4-mm long pieces.

#### 4.2.2. Conservation of Aortic Rings and Experimental Groups

As previously described [35,36,37] the aortic rings were placed in test tubes containing physiological saline solution supplemented with either dimethyl sulfoxide (DMSO at 0.5%) vehicle (IR group, n = 28–40 rings, 8–10 rats) or 50 µM CANA (IR + CANA group (n = 18–43 rings, 9–11 rats) and stored for 24 h at 4 °C. To extrude oxygen, saline solution was previously gassed with nitrogen to adjust the partial pressure of oxygen (pO_2_) to 70–74 mmHg. After cold ischemic conservation, the rings were mounted in organ baths. To mimic free radical generation and endothelial dysfunction, as occur during reperfusion and reoxygenation *in vivo*, sodium hypochlorite (200 µM, 30 min) was added to the tissue chambers. Aortic rings in the control group (n = 26–40 rings, 9–10 rats) were immediately mounted in isolated tissue baths after preparation without cold ischemic storage and hypochlorite incubation. In the present study, 50 µM canagliflozin was used, as the concentrations of 10–50 µM reflects the clinical C_max_ (10 µM) employed in the animal experiments [5].

#### 4.2.3. *Ex Vivo* Organ Baths Functional Experiments

As previously reported [38,39,40], each aortic ring was mounted on a stainless steel hook in an organ chamber containing 20 mL of KHL and continuously gassed with a mixture of 95% O_2_–5% CO_2_, with temperature maintained at 37 °C and pH at 7.4 (EMKA Technologies S.A.S, Paris, France). Initially, the tissue was equilibrated for 20 min at a resting tension before any experimental intervention. During an additional equilibration period of 60 min, the passive tension was adjusted periodically to 2 g during which the baths were rinsed with fresh KHL every 30 min as a precaution against interfering metabolites. At the beginning of each experiment, a precontraction was achieved by adding KCl (80 mM) to organ baths to ensure tissue viability and to prepare the rings for stable contractions. After the contractile response had stabilized for approximately 30 min, aortic rings were washed until resting tension was again restored. Then, the rings were contracted with an α-adrenergic receptor agonist, PE (10^−9^–10^−5^ M) until a stable plateau was reached, and the relaxation responses were investigated by adding cumulative concentrations of endothelium-dependent vasorelaxant ACh (10^−9^–10^−4^ M). For testing relaxing responses of smooth muscle cells, an endothelium-independent dilator SNP (10^−10^–10^−5^ M) was used in PE (10^−6^ M)-precontracted aortic rings. Relaxation was expressed as a percent of the contraction induced by PE. Half-maximal response (EC_50_) to PE, ACh, or SNP were determined from each individual concentration-response curve by sigmoidal fits using Origin 7.0 (Microcal Software, Northampton, Massachusetts, USA). The sensitivity pD_2_ (−logEC_50_) was then calculated.

### 4.3. Gene Expression Analysis

Using an RT^2^ Profiler PCR Array (Qiagen, Hilden, Germany), the expression of 88 genes involved in inflammation, apoptosis, and oxidative stress was profiled. The list of genes is presented in Table 3. As previously reported [41,42], total RNA was extracted from aorta samples using RNeasy Mini Kit (Qiagen, Hilden, Germany) and was reverse-transcribed into cDNA using the RT^2^ First Strand Kit, mixed with RT^2^ qPCR Master Mix containing SYBR Green, according to the manufacturer’s instructions (Qiagen, Hilden, Germany). In this custom array, the following nonregulated genes (genes of interest) were used for normalization in the fold change expression data calculations: ribosomal protein lateral stalk subunit P1 (*Rplp1*), actine beta (*ACTB*), beta-2 microglobulin (*B2m*), hypoxanthine-guanine phosphoribosyl transferase-1 (*Hprt1*), and lactate dehydrogenase A (*Ldha*). Genes at *p* < 0.05 with fold regulation values greater than 2 or less than −2 indicate increased or decreased mRNA expression, respectively, and were considered significantly altered.

### 4.4. Machine Learning Algorithms

We used Boruta [43], a machine-learning method based on permutated random forest to identify non-linear correlations between gene expression and the study subgroups (Control/IR/IR + CANA). CrossBoruta uses the split probability (0–100%) that an ensemble of decision trees will identify a specific pattern, and the “feature importance” (which resembles the effect size in traditional statistics) in combination with a stress majorization graph [44]. This method permits the exploration of nonlinear correlations across all genes, explaining the ability to reveal the underlying gene–gene interaction network. Finally, in our graph, the Spearman correlation is color-coded.

### 4.5. Immunohistochemical Staining for ICAM-1, PECAM-1, Nitrotyrosine, and Caspase-3

Immunohistochemistry was performed on buffered paraformaldehyde solution (4%) fixed, paraffin embedded, distal regions of the aortic segments. Four-µm thick sections were cut with the Leica microtome (Leica Biosystems Nussloch GmbH, Nussloch, Germany) and placed on slides. Fifteen minutes of hydrogen peroxide (3%) was used to prevent the endogenous peroxidase activity. To unmask the antigenic epitopes, the sections were pretreated in sodium citrate buffer (pH = 6) for 20 min by heating in a microwave oven at 700 Watt, then blocked in 2% normal serum for 30 min at room temperature. After that, the sections were incubated overnight at 4 °C with mouse monoclonal IgG anti-ICAM-1 (1:100; abcam, Cambridge, UK), rabbit monolyclonal IgG anti-PECAM-1 (1:10,000; abcam, Cambridge, UK), mouse monolyclonal IgG2b anti-nitrotyrosine (1:1000; abcam, Cambridge, UK), and rabbit polyclonal IgG anti-caspase-3 (1:100; antibodies-online GmbH, Aachen, Germany) antibodies. The samples were then incubated for 30 min with a biotinylated secondary antibody diluted in serum buffer (1:50), and immunoreactivity was visualized using an avidin-biotinylated complex (ABC) reagent (VECTASTAIN universal elite ABC kit, Burlingame, CA, USA). Next, 3,3′ diaminobenzidine (DAB substrate) was added to produce a brown-colored reaction product in the presence of horseradish peroxidase enzyme and used in double labeling applications (VECTOR DAB kit, Burlingame, CA, USA). During the last step, slide sections were cleared before mounting with ProTaqs Mount Aqua (Quartett, Berlin, Germany) and counterstained with haematoxylin. Digital images were captured using a conventional light microscope. Semiquantitative immunohistochemical analysis was performed with CellSens software (Olympus Soft Imaging Solutions GmbH, Germany) based on staining distribution patterns (along the endothelium for ICAM-1 and PECAM-1 or smooth muscle tissue for nitrotyrosine) and the intensity of the staining (score: 0–12). Nitrotyrosine content was determined by quantitative analysis using automated scoring with Image J plug-in IHC profiler. Evaluation from four randomized nonoverlapping fields of the aorta was done in a blinded fashion.

### 4.6. Canagliflozin

Canagliflozin 10 mM (1 mL in DMSO) was bought from Selleck Chemicals (BIOZOL Diagnostica Vertrieb GmbH, Eching, Germany) and used at a final concentration of 50 µM.

### 4.7. Data Analysis and Statistics

The results are presented as the mean ± standard error of the mean (SEM). Statistical analyses were performed using GraphPad Prism 7.02 software (GraphPad Software, Inc., CA, USA). For contractile responses to KCl and histological results. the Shapiro–Wilk normality test was used to assess normal distribution before statistical tests were applied. For data with normal distribution, one-way ANOVA and Tukey’s post-hoc test were carried out for multiple comparisons. If the data were not normally distributed, the nonparametric Kruskal–Wallis test was used, followed by Dunn’s post-hoc test. In cases of cumulative concentration-response curves to PE, Ach and SNP, a two-factor mixed ANOVA (factors: CANA and concentration of reagents (PE, ACh, SNP)) and a Tukey’s post-hoc test were used for multiple comparisons. A *p* < 0.05 was considered statistically significant. For the PCR array gene expression, *p* values were calculated using a Student’s t-test (two-tail distribution and equal or unequal variances between the two samples) with the average deltaCt value. Genes with arbitrary fold regulation cut-offs of >2-fold or <−2-fold at adjusted *p* < 0.05 were considered to be significantly altered.

## 5. Conclusions

The addition of CANA to the preservation solution alleviates endothelial dysfunction following *in vitro* IRI in aortic rings from nondiabetic rats. CANA can exert its protective effects independent of glucose lowering and leucocyte engagement, at least in part, by lowering inflammatory response through reducing the expression of inflammatory molecule ICAM-1, increasing the expression of the endothelial marker PECAM-1 and the downregulation of proinflammatory genes Il1a, Il6a, NoxO1 and by preventing the upregulation of Cd40. Our machine learning approach predicted that Il1a, Il6a, cd40 and Icam1 may be modified by CANA. From a clinical point of view, in patients undergoing CABG, IRI is the main contributor to tissue damage. It is tempting to speculate that the current study raises the possibility of repurposing this clinically approved antidiabetic drug, to be used during bypass surgery graft storage.

## Figures and Tables

**Table 1:** Quantitative analysis of vascular function after ischemia/reperfusion (IR) injury. Data are represented as mean ± SEM. CANA indicates canagliflozin; PE, phenylephrine as the percentage of the maximum contraction induced by potassium chloride (KCl); ACh, acetylcholine; SNP, sodium nitroprusside; R_max_, maximal relaxation, and pD_2_, negative logarithm of the corresponding half-maximal response (EC_50_). * *p* < 0.05 versus Control; ^#^ *p* < 0.05 versus IR. n = 18–43 aortic rings from 8–11 rats.

	Control	IR	IR + CANA
PE (% of KCl)	2.6 ± 0.2	2.5 ± 0.1	1.9 ± 0.1 *^,#^
pD_2_ to PE	6.77 ± 0.07	7.20 ± 0.14 *	6.84 ± 0.06 ^#^
KCl (g)	4.6 ± 0.2	1.2 ± 0.1 *	2.1 ± 0.1 *^,#^
R_max_ to ACh (%)	75.2 ± 2.3	31.7 ± 3.2 *	51.9 ± 2.5 *^,#^
pD_2_ to ACh	7.35 ± 0.08	6.28 ± 0.15 *	6.86 ± 0.09 *^,#^
R_max_ to SNP (%)	99.4 ± 0.2	99.0 ± 0.6	100.0 ± 0.0
pD_2_ to SNP	8.65 ± 0.06	7.97 ± 0.07 *	8.49 ± 0.09 ^#^

**Table 2:** Gene expression, including the *p*-values and fold regulation of all genes tested. For statistically significant genes, fold-regulation values are presented in bold. See Table 1 for gene abbreviations. “A” means that this gene’s average threshold cycle is relatively high (>30) in either the control or the test sample and is reasonably low in the other sample (<30), “B” means that this gene’s average threshold cycle is relatively high (>30) (meaning that its relative expression level is low in both control and test samples, and the *p*-value for the fold-change is either unavailable or relatively high (*p* > 0.05)), and “C” means that this gene’s average threshold cycle is either not determined or greater than the defined cut-off value in both samples (meaning that its expression was undetected, making this fold-change result erroneous and uninterpretable). IR indicates ischemia/reperfusion and CANA canagliflozin.

IR vs. Control				IR + CANA vs. Control				IR + CANA vs. IR			
Symbol	*p*-Value	Regulation	Comments	Symbol	*p*-Value	Regulation	Comments	Symbol	*p*-Value	Regulation	Comments
*Aifm1*	0.527	1.06		*Aifm1*	0.277	1.09		*Aifm1*	0.764	1.03	
*Apaf1*	0.716	1.03		*Apaf1*	0.361	1.06		*Apaf1*	0.67	1.04	
*Bad*	0.059	−1.17		*Bad*	0.894	−1.01		*Bad*	0.05	1.16	
*Bak1*	0.374	−1.1		*Bak1*	0.532	−1.07		*Bak1*	0.545	1.03	
*Bax*	0.023	1.1		*Bax*	0.168	1.06		*Bax*	0.259	−1.04	
*Bcl2*	0.123	1.06		*Bcl2*	0.791	−1.00		*Bcl2*	0.071	−1.06	
*Bcl2L1*	0.321	1.09		*Bcl2L1*	0.453	1.08		*Bcl2L1*	0.919	−1.01	
*Bid*	0.068	1.83		*Bid*	0.252	1.41		*Bid*	0.041	−1.29	
*Casp1*	0.018	−1.28		*Casp1*	0.061	−1.27		*Casp1*	0.945	1.01	
*Casp12*	0.055	1.18		*Casp12*	0.229	1.11		*Casp12*	0.241	−1.07	
*Casp3*	0.256	1.1		*Casp3*	0.247	1.11		*Casp3*	0.91	1.01	
*Casp4*	0.146	1.18		*Casp4*	0.156	1.19		*Casp4*	0.814	1.01	
*Casp6*	0.276	1.17		*Casp6*	0.863	−1.03		*Casp6*	0.191	−1.21	
*Casp7*	0.607	1.06		*Casp7*	0.026	1.26		*Casp7*	0.128	1.19	
*Casp8*	0.461	1.08		*Casp8*	0.059	1.20		*Casp8*	0.266	1.1	
*Casp9*	0.74	−1.03		*Casp9*	0.44	1.07		*Casp9*	0.272	1.1	
*Cat*	0.767	1.14	B	*Cat*	0.818	−1.10	B	*Cat*	0.581	−1.25	B
*Ccl11*	0.171	1.64		*Ccl11*	0.841	1.07		*Ccl11*	0.246	−1.52	
*Ccl12*	0	**8.55**		*Ccl12*	0	**10.09**		*Ccl12*	0.658	1.18	
*Ccl2*	0	**24.21**		*Ccl2*	0	**21.75**		*Ccl2*	0.53	−1.11	
*Ccl20*	0.787	−1.01	C	*Ccl20*	0.137	−1.08	C	*Ccl20*	0.089	−1.06	C
*Ccl3*	0.003	**71.33**		*Ccl3*	0.003	**75.18**		*Ccl3*	0.73	1.05	
*Ccl4*	0	**34.85**		*Ccl4*	0	**36.45**		*Ccl4*	0.806	1.05	
*Ccl5*	0.011	**−2.81**		*Ccl5*	0.012	**−2.82**		*Ccl5*	0.985	−1	
*Ccr1*	0.153	−4.21		*Ccr1*	0.028	−1.65		*Ccr1*	0.326	2.55	
*Ccr2*	0.461	−1.18		*Ccr2*	0.776	−1.05		*Ccr2*	0.556	1.12	
*Ccs*	0.113	−1.22		*Ccs*	0.163	−1.1		*Ccs*	0.349	1.11	
*Cd40*	0.004	**2.13**		*Cd40*	0.04	1.6		*Cd40*	0.206	−1.34	
*cd40lg*	0.281	−1.56	B	*cd40lg*	0.882	−1.07	B	*cd40lg*	0.428	1.46	B
*Cflar*	0.894	1.01		*Cflar*	0.675	1.02		*Cflar*	0.885	1.01	
*CxCr4*	0	**4.47**		*CxCr4*	0	**3.37**		*CxCr4*	0.144	−1.32	
*Cyba*	0.092	1.07		*Cyba*	0.281	1.1		*Cyba*	0.706	1.03	
*cycs*	0.825	1.02		*cycs*	0.972	1		*cycs*	0.807	−1.01	
*Duox1*	0.998	−1	B	*Duox1*	0.666	−1.1	B	*Duox1*	0.798	−1.09	B
*Edn1*	0.002	**2.44**		*Edn1*	0.017	**2.06**		*Edn1*	0.556	−1.19	
*Epx*	0.746	1.06		*Epx*	0.587	1.1		*Epx*	0.806	1.04	
*Fadd*	0.954	−1		*Fadd*	0.955	−1		*Fadd*	0.978	1	
*Fas*	0.973	−1.01	B	*Fas*	0.081	−1.87	B	*Fas*	0.06	−1.85	B
*Faslg*	0.926	1.02		*Faslg*	0.932	−1.02		*Faslg*	0.877	−1.05	
*Flt1*	0.143	−1.22		*Flt1*	0.242	−1.09		*Flt1*	0.352	1.12	
*Fos*	0	**51.6**		*Fos*	0	**54.88**		*Fos*	0.348	1.06	
*Gpx1*	0.738	1.02		*Gpx1*	0.065	1.11		*Gpx1*	0.054	1.09	
*Gpx2*	0.279	2.01	B	*Gpx2*	0.144	2.12	B	*Gpx2*	0.922	1.06	B
*Gpx3*	0.696	1.05		*Gpx3*	0.909	1.01		*Gpx3*	0.678	−1.04	
*Gpx4*	0.697	1.02		*Gpx4*	0.748	1.01		*Gpx4*	0.897	−1	
*Gpx5*	0.428	−1.09	B	*Gpx5*	0.198	−1.17	B	*Gpx5*	0.089	−1.06	C
*Gpx6*	0.787	−1.01	C	*Gpx6*	0.137	−1.08	C	*Gpx6*	0.089	−1.06	C
*Gpx7*	0.647	1.03		*Gpx7*	0.286	1.08		*Gpx7*	0.453	1.05	
*Gstk1*	0.305	1.11		*Gstk1*	0.453	1.07		*Gstk1*	0.655	−1.04	
*Hspa1a*	0	**4.63**	A	*Hspa1a*	0	**5.65**	A	*Hspa1a*	0.376	1.22	
*Icam1*	0	**4.34**		*Icam1*	0	**3.66**		*Icam1*	0.233	−1.19	
*Il10*	0	**35.06**	A	*Il10*	0	**38.69**	A	*Il10*	0.63	−1.1	
*Il18*	0.817	1.03		*Il18*	0.316	−1.16		*Il18*	0.306	−1.2	
*Il1a*	0	**210.71**	A	*Il1a*	0	**138.48**	A	*Il1a*	0.193	−1.52	
*Il1b*	0	**28.95**		*Il1b*	0	**24.13**		*Il1b*	0.386	−1.2	
*Il6*	0	**95.21**		*Il6*	0	**53.87**		*Il6*	0.086	−1.77	
*Il7*	0.238	−1.38		*Il7*	0.693	1.08		*Il7*	0.099	1.48	
*Il9*	0.381	−1.22	B	*Il9*	0.659	−1.12	B	*Il9*	0.591	1.09	B
*Itgb2*	0.028	−1.44		*Itgb2*	0.015	−1.55		*Itgb2*	0.668	−1.08	
*Jun*	0.005	1.34		*Jun*	0.027	1.31		*Jun*	0.813	−1.02	
*Ncf1*	0.827	1.03		*Ncf1*	0.136	−1.12		*Ncf1*	0.247	−1.16	
*Nfkb1*	0.046	1.11		*Nfkb1*	0.074	1.11		*Nfkb1*	0.96	−1	
*Nos2*	0.891	−1.08	B	*Nos2*	0.686	1.29	B	*Nos2*	0.567	1.39	B
*Nox4*	0.023	1.35		*Nox4*	0.372	1.11		*Nox4*	0.14	−1.21	
*Noxo1*	0.127	−1.9	B	*Noxo1*	0.036	**−2.27**	B	*Noxo1*	0.674	−1.2	B
*Prdx1*	0.239	1.12		*Prdx1*	0.376	1.09		*Prdx1*	0.178	−1.03	
*Prdx2*	0.01	1.23		*Prdx2*	0.026	1.21		*Prdx2*	0.593	−1.02	
*Prdx3*	0.099	1.19		*Prdx3*	0.088	1.21		*Prdx3*	0.841	1.01	
*Prdx4*	0.179	1.09		*Prdx4*	0.027	1.14		*Prdx4*	0.49	1.05	
*Sele*	0	**8.3**	A	*Sele*	0	**9.81**	A	*Sele*	0.644	1.18	
*Slc5a1*	0.241	−1.23	B	*Slc5a1*	0.847	−1.06	B	*Slc5a1*	0.516	1.16	B
*Sod1*	0.352	−1.09		*Sod1*	0.948	−1.01		*Sod1*	0.234	1.09	
*Sod2*	0.005	1.32		*Sod2*	0.001	1.33		*Sod2*	0.991	1	
*Sod3*	0	1.24		*Sod3*	0.002	1.36		*Sod3*	0.217	1.1	
*Tgfb1*	0.129	1.11		*Tgfb1*	0.176	1.09		*Tgfb1*	0.764	−1.02	
*Tlr1*	0.082	−1.49		*Tlr1*	0.183	−1.27		*Tlr1*	0.468	1.18	
*Tlr4*	0.83	1.03		*Tlr4*	0.284	1.19		*Tlr4*	0.267	1.16	
*Tlr6*	0.095	−1.23		*Tlr6*	0.131	−1.16		*Tlr6*	0.575	1.06	
*Tnf*	0	**17.56**		*Tnf*	0	**14.7**		*Tnf*	0.483	−1.19	
*Tnfrsf12a*	0.947	1.01		*Tnfrsf12a*	0.609	−1.1		*Tnfrsf12a*	0.462	−1.12	
*Tollip*	0.06	1.1		*Tollip*	0.027	1.2		*Tollip*	0.204	1.1	
*Tp53*	0.051	3.7	B	*Tp53*	0.076	4.79	B	*Tp53*	0.762	1.3	B
*Txn1*	0.202	1.05		*Txn1*	0.386	1.03		*Txn1*	0.437	−1.02	
*Txnrd1*	0	1.28		*Txnrd1*	0	1.34		*Txnrd1*	0.325	1.05	
*Txnrd2*	0.428	1.04		*Txnrd2*	0.773	1.02		*Txnrd2*	0.772	−1.02	
*Vcam1*	0.011	1.73		*Vcam1*	0.024	1.75		*Vcam1*	0.934	1.01	
*Vegfc*	0.156	1.24		*Vegfc*	0.154	1.26		*Vegfc*	0.87	1.02	
*Xiap*	0.381	1.1		*Xiap*	0.14	1.21		*Xiap*	0.116	1.09	

**Table 3:** Gene symbol and name.

Name/Gene ID/Symbol	Gene Name/Description (*for* Rattus Norvegicus (*=Wistar rat*))
*Aifm1*	apoptosis inducing factor, mitochondria associated 1
*Apaf1*	apoptotic peptidase activating factor 1
*Bad*	BCL2-associated agonist of cell death
*Bak1*	BCL2-antagonist/killer 1
*Bax*	BCL2 associated X, apoptosis regulator
*Bcl2*	*BCL2*, apoptosis regulator
*Bcl2L1*	Bcl2-like 1
*Bid*	BH3 interacting domain death agonist
*Casp1*	caspase 1
*Casp12*	caspase 12
*Casp3*	caspase 3
*Casp4*	caspase 4
*Casp6*	caspase 6
*Casp7*	caspase 7
*Casp8*	caspase 8
*Casp9*	caspase 9
*Cat*	catalase
*Ccl11*	C-C motif chemokine ligand 11
*Ccl12*	chemokine (C-C motif) ligand 12
*Ccl2*	C-C motif chemokine ligand 2
*Ccl20*	C-C motif chemokine ligand 20
*Ccl3*	C-C motif chemokine ligand 3
*Ccl4*	C-C motif chemokine ligand 4
*Ccl5*	C-C motif chemokine ligand 5
*Ccr1*	C-C motif chemokine receptor 1
*Ccr2*	C-C motif chemokine receptor 2
*Ccs*	copper chaperone for superoxide dismutase
*Cd40*	*CD40* molecule
*cd40lg*	CD40 ligand
*Cflar*	CASP8 and FADD-like apoptosis regulator
*CxCr4*	C-X-C motif chemokine receptor 4
*Cyba*	cytochrome b-245 alpha chain
*cycs*	cytochrome c, somatic
*Duox1*	dual oxidase 1
*Edn1*	endothelin 1
*Epx*	eosinophil peroxidase
*Fadd*	Fas associated via death domain
*Fas*	Fas cell surface death receptor
*Faslg*	Fas ligand
*Flt1*	Fms related receptor tyrosine kinase 1
*Fos*	*Fos* proto-oncogene, AP-1 transcription factor subunit
*Gpx1*	glutathione peroxidase 1
*Gpx2*	glutathione peroxidase 2
*Gpx3*	glutathione peroxidase 3
*Gpx4*	glutathione peroxidase 4
*Gpx5*	glutathione peroxidase 5
*Gpx6*	glutathione peroxidase 6
*Gpx7*	glutathione peroxidase 7
*Gstk1*	glutathione S-transferase kappa 1
*Hspa1a*	heat shock protein family A (Hsp70) member 1A
*Icam1*	intercellular adhesion molecule 1
*Il10*	interleukin 10
*Il18*	interleukin 18
*Il1a*	interleukin 1 alpha
*Il1b*	interleukin 1 beta
*Il6*	interleukin 6
*Il7*	interleukin 7
*Il9*	interleukin 9
*Itgb2*	integrin subunit beta 2
*Jun*	Jun proto-oncogene, AP-1 transcription factor subunit
*Ncf1*	neutrophil cytosolic factor 1
*Nfkb1*	nuclear factor kappa B subunit 1
*Nos2*	nitric oxide synthase 2
*Nox4*	NADPH oxidase 4
*Noxo1*	NADPH oxidase organizer 1
*Prdx1*	peroxiredoxin 1
*Prdx2*	peroxiredoxin 2
*Prdx3*	peroxiredoxin 3
*Prdx4*	peroxiredoxin 4
*Sele*	selectin E
*Slc5a1*	solute carrier family 5 member 1
*Sod1*	superoxide dismutase 1
*Sod2*	superoxide dismutase 2
*Sod3*	superoxide dismutase 3
*Tgfb1*	transforming growth factor, beta 1
*Tlr1*	toll-like receptor 1
*Tlr4*	toll-like receptor 4
*Tlr6*	toll-like receptor 6
*Tnf*	tumor necrosis factor
*Tnfrsf12a*	TNF receptor superfamily member 12A
*Tollip*	toll interacting protein
*Tp53*	tumor protein p53
*Txn1*	thioredoxin 1
*Txnrd1*	thioredoxin reductase 1
*Txnrd2*	thioredoxin reductase 2
*Vcam1*	vascular cell adhesion molecule 1
*Vegfc*	vascular endothelial growth factor C
*Xiap*	X-linked inhibitor of apoptosis

## Data Availability

Data associated with this study is available upon reasonable request.
